# Forensic investigation on a combined death by food aspiration and acute escitalopram intoxication occurred to a psychiatric subject in a nursing home

**DOI:** 10.1007/s00414-024-03168-5

**Published:** 2024-02-06

**Authors:** P. Zuccarello, G. Carnazza, M. Salerno, M. Esposito, S. Cosentino, A. Giorlandino, F. Sessa, C. Pomara, N. Barbera

**Affiliations:** 1 Department of Medical, Surgical and Advanced Technologies “G.F. Ingrassia, University of Catania, Catania, Italy; 2 Department of Pathological Anatomy, A.R.N.A.S. Garibaldi-Nesima, Catania, Italy

**Keywords:** Combined death, Food aspiration, Escitalopram, Psychiatry, Forensic, Nursing

## Abstract

Food aspiration is one of the major health risks for elderly people in nursing homes which could lead to death. Moreover, misconducts in pharmacotherapy may represent a potential risk of adverse drug reactions. It is reported here the toxicological evaluation of a combined death by food aspiration and acute escitalopram intoxication of a psychiatric subject, occurred in a nursing home. An 89-year-old man, suffering from dysphagia and Alzheimer’s, was resident in a nursing home. He was fed with a liquid diet administered directly in mouth using a syringe. The man was also being treated with escitalopram 10 mg tablet. One evening, after receiving the meal in the usual way, the man complained of sudden illness. Carried to the emergency room, the man died about 3 h later with a diagnosis of cardiogenic shock subsequentially to ab ingestis. The histological findings revealed the presence of exogenous material, probably food, up to the finest bronchial branches. The toxicological examination revealed the presence of escitalopram and its main metabolite, desmethylcitalopram: in the blood 1972 ng/ml and 285 ng/ml, in the brain 4657 ng/g and 1025 ng/g, in the gastric content 2317 ng/g and 423 ng/g, in the lung 21,771 ng/g and 468 ng/g, respectively. The bad practice of the nurses to dissolve the escitalopram tablet in the liquefied food and to administer the therapy with a syringe directly into the mouth emerged thanks this investigation. Following food aspiration, escitalopram was absorbed by inhalation route, reaching high concentrations in blood and tissues. The death occurred due to a combined mechanism between food aspiration and the escitalopram toxic action.

## Introduction

Pulmonary aspiration is one of the major health risks for elderlies in nursing home (NH), which could lead to respiratory infections, aspiration pneumonia or sudden bolus death [1]. Dysphagia, neurological disorders, and drug-sedation comprise the commonest risk factors for aspiration-related deaths [2].

Over the years, there has been an increase in use of antidepressant in NH [3], especially among residents with dementia or cognitive impairment [4]. In NH, misconducts in medication therapy may represent a potential risk of adverse drug reactions and accidental poisonings [5].

Medical liability is an increasingly issue worldwide; however, few studies are still reported to demonstrate when the medical malpractice occurred [6].

The toxicologist can provide an important contribution to the forensic investigation in establishing the pathogenetic mechanism underlying the death but also in identifying any medical malpractice from a pharmacological point of view.

It is reported here the toxicological evaluation of a combined death by food aspiration and acute escitalopram intoxication occurred in a nursing home.

## Material and method

### Case history

An 89-year-old man, suffering from dysphagia and Alzheimer’s, was resident in a nursing home. He was also suffering from hypercholesterolemia, urinary and fecal incontinence, carotid atherosclerosis, gastroesophageal reflux, hiatal hernia, and bedrest.

During his stay in the nursing home, several times, he was transported to the hospital for repeated episodes of cerebral ischemia.

Due to his dysphagia, he was fed with a liquid diet administered directly in mouth using a syringe. The man was being treated with 10 mg/day escitalopram tablet.

One evening, after receiving the meal in the usual way, the man complained of sudden illness, with vomiting and breathing difficulties, a SatO2 of 72%, hypotension, and tachycardia. Carried to the emergency room, he arrived in a soporous-comatose state, with rales, undetectable saturation, and arterial pulses present.

The man died about 3 h later with a diagnosis of cardiogenic shock from suspected ab ingestis with acute respiratory failure in a patient with senile dementia.

At first, the autopsy and histopathological examinations were disposed. A complete autopsy was performed about 24 h after death. Samples for possible further toxicological investigation were also collected, that were stored at − 20 °C.

To evaluate if excessive drug doses were administered to the man, such as to alter his psychophysical state at the time of aspiration, the forensic toxicological investigation was also subsequently ordered.

### Histopathological analysis

Histological sections of the heart, lungs, kidneys, liver, and brain were stained with hematoxylin and eosin for microscopic examination [7].

### Toxicological analysis

Complete toxicological investigation was performed. Untarget analysis, ethanol, common abuse substance, benzodiazepines, antidepressant, and antipsychotic were analyzed on femoral blood sample.

For analyses of escitalopram and desmethylcitalopram, aliquots of 2 ml of femoral blood and 2 g of homogenated brain, lung, and gastric content samples were extracted by LLE method [8]. Each sample was added with clozapine (internal standard) at 1000 ng/ml or ng/g. Subsequently, each sample was added with 4 ml of saturated sodium solution (pH = 11.5) and 1.5 g of sodium chloride. After, the mixture was extracted twice with chlorobutane (8 ml and 4 ml of solvent, respectively).

The organic phases were taken, collected in clean tubes, and added with 3 ml of sulfuric acid 0.1 N. Then, after the agitation and centrifugation, the water phase of each sample was taken and extracted twice with 2.5 ml of chlorobutane, at first at pH = 8, by alkalization with 0.5 ml of phosphate buffer at pH = 7 and 0.5 ml of sodium hydroxide 1 N, and subsequently to pH 12 by a further addition of sodium hydroxide 1 N. For of each sample, the two organic phases have been collected together and the solvent was evaporated by nitrogen flow up to obtain dried residues; these have been solubilized with 50 µl of acetone and transferred to autosampler vials with conical insert, for the followed GC–MS analyses.

The samples were analyzed by a gas chromatograph from Agilent Technologies (AT) mod. 6890N connected to an AT mass spectrometer mod. 5973 Inert with AT 7683 Series autosampler.

The chromatographic run was conducted at a programmed temperature: initially the temperature varied from 100 to 220 °C with increments of 30 °C per minute and from 220 to 300 °C with increments of 5 °C per minute; finally, it is kept constant for 3 min at 300 °C. The capillary column used is an Agilent EVDX-5MS, 25-m long, 0.20 mm i.d., and 0.33-mcm film thickness.

The amount of sample injected into the gas chromatograph was 1 µl. The detection was performed in Selected Ions Monitoring (SIM) mode. Table 1 schematizes the ions of escitalopram, desmethylcitalopram, and clozapine.

**Table 1:** SIM parameters of escitalopram, desmethylcitalopram, and clozapine (I.S.)

Analytes	First ion	Second ion	Third ion
*Escitalopram*	58	238	208
*Desmethylcitalopram*	44	238	310
*Clozapina (I.S.)*	243	256	192

## Results

**Fig. 1:**
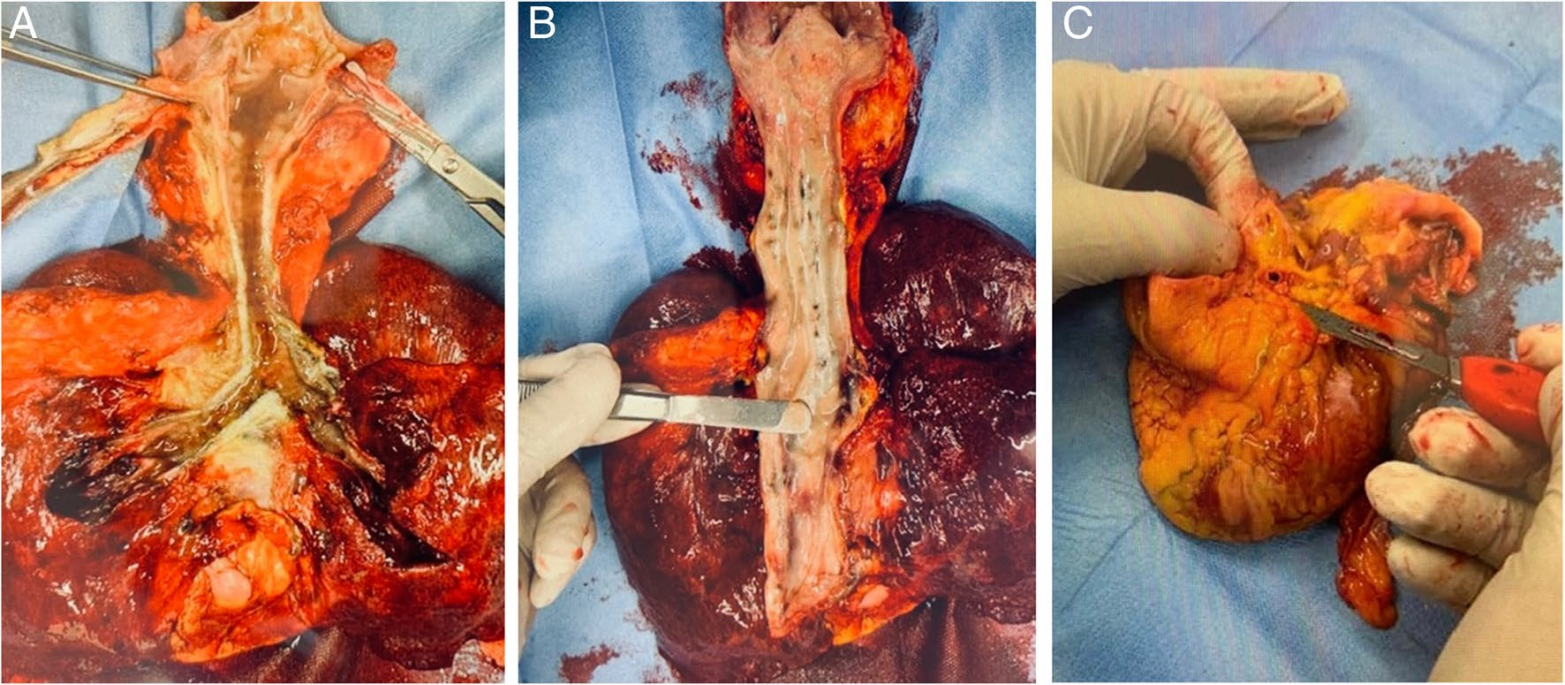
Fluid in the trachea and bronchia (**A**), exogenous material up to the bronchial branches (**B**), back surface of the heart, and coronary section (**C**)

The autopsy mainly ascertained: pleural adhesions at the apex of the left lung; fluid blood without thrombus and clots into the pulmonary vessels; a massive amount of foam into the trachea and the lungs; exogenous material, probably food, up to the bronchial branches (Fig. 1). The weight and the height of the body and organs weight are reported in Table 2.

**Table 2:** Weight and height of the body and organs weight

	Units	Measurements
*Body height*	cm	139
*Body weight*	kg	53
*Left lung weight*	g	356
*Right lung weight*	g	325
*Brain weight*	g	1029
*Liver weight*	g	587
*Left kidney weight*	g	68
*Right kidney weight*	g	74
*Heart weight*	g	272

**Fig. 2:**
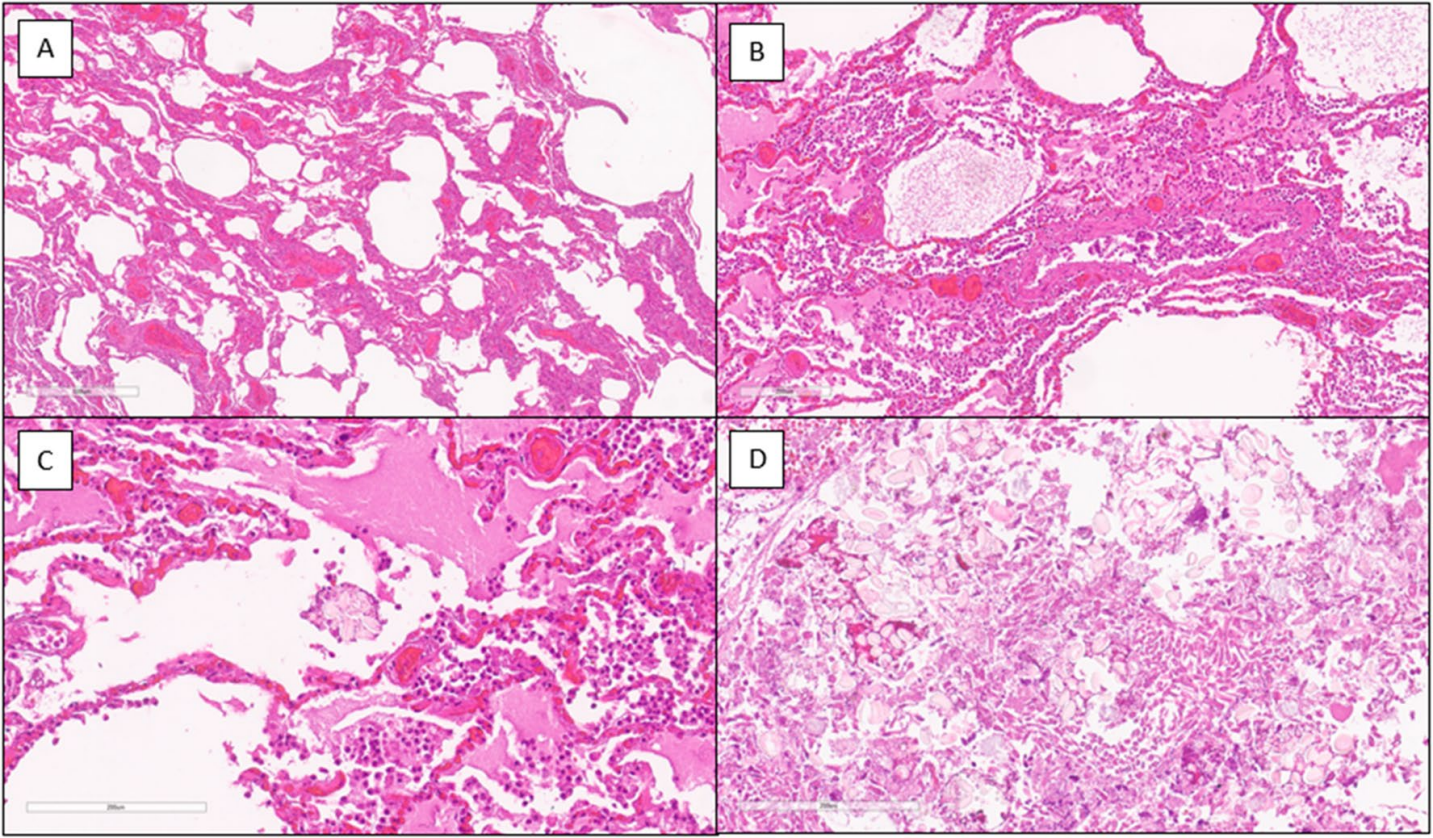
Dilation of the respiratory spaces alternating with endoalveolar edema and pulmonary vasculopathy [(**A**, **B**), H&E]. Foci of bronchopneumonia and presence of exogen material (**C**, H&E) compatible with liquified food, up to the finest bronchial branches (**D**, H&E)

**Fig. 3:**
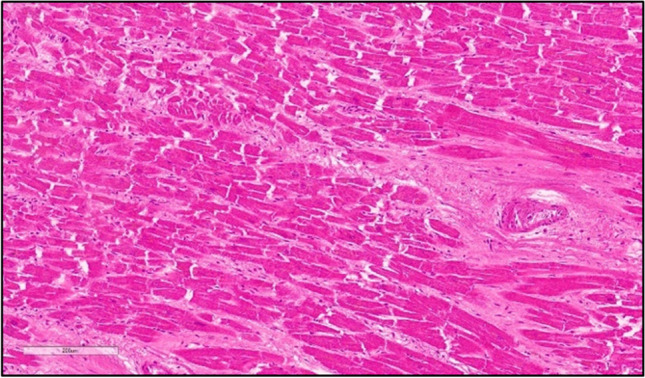
Hypertrophy of cardiomyocytes associated with fine interstitial scars and small vessels obstructive disease (H&E)

The histological findings of the lungs revealed a dilation of the respiratory spaces, alternating with bronchopneumonia foci, and the presence up to the finest bronchial branches of exogenous material, compatible with liquified food. In addition, there were cases of endo-alveolar hemorrhage, diffuse arteriolar micro-thromboembolism, and endo-alveolar edema with pulmonary vasculopathy (Fig. 2). On the heart, a myocyte hypertrophy associated with fine interstitial fibrosis, small interstitial scars (Fig. 3), moderate coronary occlusion due to the histopathologic picture of a chronic ischemic heart disease and hypertrophy of the right ventricular myocardium were showed.

The histological findings of the brain revealed a widespread hydropic degeneration without evidence of major ischemic injury. The liver showed preserved laminar architecture and marked congestion of the sinusoidal network. Kidneys showed histological findings of interstitial nephritis with hyalinosis of multiple glomeruli.

The toxicological examination revealed the presence of escitalopram and its main metabolite, desmethylcitalopram in all investigated samples. In Table 3, femoral blood, brain, lung and, finally, gastric content concentrations of escitalopram and its metabolite are reported. No other substance was highlighted.

**Table 3:** Escitalopram and desmethylcitalopram concentrations

Samples	Units	Escitalopram	Desmethylcitalopram
*Femoral blood*	ng/ml	1972	285
*Brain*	ng/g	4657	1025
*Lung*	ng/g	21,771	468
*Gastric content*	ng/g	2317	423

## Discussion and conclusion

Initially, the circumstantial data, autopsy, and histopathological findings led the forensic pathologist to conclude that the death was due to an asphyxia death, subsequentially to an ab ingestis.

However, the hypothesized dynamic was not fully explanatory of the case. Aspiration of gastric material can lead to choking, pneumonitis, pneumonia, and acute respiratory distress syndrome [9]. Therefore, the aspiration could lead to or a rapid death due to airway occlusion [10] or the onset of a respiratory infection which has a slower course respect to what occurred in our case [11].

At first, the toxicological investigation was disposed only to establish whether the psychophysical conditions of the man at time of death were altered by drug action.

The toxicological analyses revealed a high concentration of escitalopram in the blood, compatible with acute or lethal intoxication.

Escitalopram is an antidepressant drug belonging to selective serotonin reuptake inhibitors (SSRIs). It is considered a rather safe drug as intoxication with fatal outcome is rare. There is no reference toxic dose [12]. Only some cases of escitalopram intoxication, even lethal, are reported in the literature [13]. Postmortem escitalopram blood concentrations about 360 ng/mL could be considered dangerous [14].

The hypothesis of the toxicologist was that the death occurred due to a combined mechanism between the aspiration of food and the toxic action of escitalopram, which probably led quickly to arrhythmias, respiratory distress, and comatose state [15]. Given the high concentrations of escitalopram mainly in the lung, it was also suspected that the tablet was dissolved in liquefied food administered by a syringe that was accidentally aspirated. The bioavailability of the drug absorbed from the respiratory tract is far greater compared to the oral route. The inhalation route consists of a large absorption surface represented by the pulmonary alveoli which are in very close contact with the blood capillaries; subsequentially, a rapid and high absorption of the inhaled substances derives. Furthermore, the bioavailability of the inhalation route also increases due to the lack of first pass hepatic metabolism [16].

This insight and toxicological data led the magistrate to gather additional information questioning the nursing staff. From their statements, it emerged that the nurses used to dissolve the escitalopram tablet in liquefied food and to administer it with a syringe directly into the mouth.

Although manipulating the pharmaceutical formulation is a very common procedure and can often provide numerous benefits [17], sometimes the practice of crushing or splitting tablets can lead to health problems, as not all tablets are suitable for this purpose, for example, film-coated escitalopram tablets [18]. The crushing of film-coated, prolonged, or extended-release tablets could compromise their efficacy, as well as increase the risk of adverse effects and acute poisoning due to the uncontrolled release of the active ingredient [17].

In cases where the patient is unable to take the tablets intact, it would be necessary to recur to liquid solutions, for example, drops. Furthermore, for dysphagia patients, it would be necessary to administer the crashed and dissolved drugs using enteral feeding tubes, such as the nasogastric tube or the Percutaneous Endoscopic Gastrostomy (PEG) [18].

Therefore, trituration of the drug, dissolution in food, and subsequent administration directly into the mouth by syringe cannot be considered suitable and safe practices. Indeed, escitalopram, reaching the deep airways, have caused an acute intoxication which, combined with aspiration, rapidly led to the death of the man.

However, some consideration about this death was highlighted. The administration of the tablet dissolved in the food preparation and the aspiration of the food into the airways justified the high concentrations of escitalopram in the blood and tissues of the man, thus excluding that an excessive dosage of the medicine was administered voluntarily or accidentally.

Moreover, since the comorbidities of which the man was affected, the food aspiration up to the finest bronchial branches would still have resulted probably in death even without the toxic action of escitalopram. Furthermore, even if the dissolved escitalopram had been in the form of drops, food aspiration would have still led the drug entering the airways.

Finally, even if the man had been fed with artificial nutrition through more appropriate medical devices such as the nasogastric tube or the PEG, there is still no scientific evidence that this practice reduces the incidence of adverse events such as accidental inhalation of food or, in any case, that it increases the survival rate in subjects with comorbidities such as dementia, persistent vegetative state [19–21].

This report could be of highest importance for healthcare professionals working in nursing homes to improve the management of psychiatric elderly patients.

Combined death is not common, but this case report underscores how critical is that the forensic pathologist should not stop to identify the most obvious first cause of death without considering the possibility of combined death mechanisms.

This report clearly highlighted the importance of the toxicologist in forensic investigations. The close collaboration between the different forensic skills is essential for the resolution of even apparently simple cases. In this investigation, without the involvement of the toxicologist, the pathogenetic mechanism of death and the bad practice of the nurses would never be discovered.

Thanks to this case report it was possible to appreciate how useful the toxicologist’s evaluation is in medico-legal investigations, not only to highlight the presence of one or more substances and their respective concentrations but also in investigating the causal link between death and any misconduct by healthcare professionals.

## Data Availability

All data are available at Forensic Toxicology Laboratories of University of Catania.

## References

[1] Chen S, Kent B, Cui Y (2021). Interventions to prevent aspiration in older adults with dysphagia living in nursing homes: a scoping review. BMC Geriatr.

[2] Hu X, Yi ES, Ryu JH (2014). Aspiration-related deaths in 57 consecutive patients: autopsy study. PLoS One.

[3] Hanlon JT, Handler SM, Castle NG (2010). Antidepressant prescribing in US nursing homes between 1996 and 2006 and its relationship to staffing patterns and use of other psychotropic medications. J Am Med Dir Assoc.

[4] Hiltunen H, Tan EC, Ilomäki J, Hilmer SN, Visvanathan R, Emery T, Robson L, Jones MJ, Hartikainen S, Bell JS (2016). Factors associated with antidepressant use in residents with and without dementia in Australian aged care facilities. Ther Adv Drug Saf.

[5] Jakobsen HN, Vermehren C, Andersen JT, Dalhoff K (2021). Drug poisoning in nursing homes: a retrospective study of data from the Danish poison information centre. Drugs Ther Perspect.

[6] Moreira H, Magalhães T, Dinis-Oliveira R, Taveira-Gomes A (2014). Forensic evaluation of medical liability cases in general surgery. Med Sci Law.

[7] Barbera N, Busardò FP, Indorato F, Bartoloni G, Romano G (2014). Fulminant ischemic colitis with a fatal outcome after cocaine snorting: case report and literature review. Egypt J Forensic Sci.

[8] Moffat AC, Osselton MD, Widdop B, Watts J (2011). Clarke’s analysis of drug and poisons.

[9] Flynn DN, Doyal A, Schoenherr JW (2022) Gastric ultrasound. StatPearls [Internet] Treasure Island (FL). https://www.ncbi.nlm.nih.gov/books/NBK580524/. Accessed 20 February 2023

[10] Duckett SA, Bartman M, Roten RA (2022) Choking. StatPearls [Internet] Treasure Island (FL). https://www.ncbi.nlm.nih.gov/books/NBK499941/#:~:text=Choking%20or%20foreign%20body%20airway,upper%20airway%20and%20the%20trachea. Accessed 20 February 2023

[11] Stapleton RD, Wang BM, Hudson LD, Rubenfeld GD, Caldwell ES, Steinberg KP (2005). Causes and timing of death in patients with ARDS. Chest.

[12] Luchini D, Morabito G, Centini F (2005). Case report of a fatal intoxication by citalopram. Am J Forensic Med Pathol.

[13] Kraai EP, Seifert SA (2015). Citalopram overdose: a fatal case. J Med Toxicol.

[14] Nishio T, Toukairin Y, Hoshi T, Arai T, Nogami M (2021). A fatal poisoning case of acetone cyanohydrin and citalopram. Leg Med (Tokyo).

[15] Jimmink A, Caminada K, Hunfeld NG, Touw DJ (2008) Clinical toxicology of citalopram after acute intoxication with the sole drug or in combination with other drugs: overview of 26 cases. Ther Drug Monit 30(3):365–71. Erratum in: (2022) Ther Drug Monit 44(2):357. 10.1097/FTD.0b013e3181379ef6 10.1097/FTD.0b013e3181379ef6 18520609

[16] Kim J, De Jesus O (2022) Medication routes of administration. StatPearls [Internet], Treasure Island (FL). https://www.ncbi.nlm.nih.gov/books/NBK568677/. Accessed 20 February 2023

[17] Quinzler R, Gasse C, Schneider A, Kaufmann-Kolle P, Szecsenyi J, Haefeli WE (2006). The frequency of inappropriate tablet splitting in primary care. Eur J Clin Pharmacol.

[18] Colechester Hospital University NHS (2012) - NEEMMC guidelines for tablet crushing and administration via enteral feeding tubes. https://www.stch.org.uk/wp-content/uploads/pct-version-neemmc-guidelines-for-tablet-crushing-april-2012.pdf. Accessed 20 February 2023.

[19] Sampson EL, Candy B, Jones L (2009) Enteral tube feeding for older people with advanced dementia. Cochrane Database Syst Rev (2):CD007209. 10.1002/14651858.CD007209 10.1002/14651858.CD007209.pub2 PMC7182132 19370678

[20] Mitchell SL, Kiely DK, Lipsitz LA (1997). The risk factors and impact on survival of feeding tube placement in nursing home residents with severe cognitive impairment. Arch Intern Med.

[21] Haddad RY, Thomas DR (2002). Enteral nutrition and enteral tube feeding. Review of the evidence. Clin Geriatr Med.

